# Pseudo-Foster Kennedy Syndrome due to unilateral optic nerve hypoplasia: a case report

**DOI:** 10.1186/1752-1947-2-86

**Published:** 2008-03-18

**Authors:** Shveta Bansal, Timothy Dabbs, Vernon Long

**Affiliations:** 1 Department of Ophthalmology, St James' University Hospital, Leeds, UK

## Abstract

**Introduction:**

Pseudo-Foster Kennedy Syndrome is described as unilateral optic disc swelling with contralateral optic atrophy in the absence of an intracranial mass causing compression of the optic nerve. This occurs typically due to bilateral sequential optic neuritis or ischaemic optic neuropathy.

**Case Presentation:**

We describe a case of pseudo-Foster Kennedy Syndrome in a two year old boy with unilateral papilloedema due to a congenital optic disc anomaly in one eye preventing transmission of raised intracranial pressure to the optic nerve.

**Conclusion:**

From our findings we conclude that congenital optic nerve hypoplasia is a cause of pseudo-Foster Kennedy Syndrome.

## Introduction

Foster Kennedy Syndrome is unilateral optic disc swelling with contralateral optic atrophy, usually due to a frontal lobe tumour compressing the optic nerve on one side and resulting in papilloedema contralaterally. In the absence of an intracranial mass these findings may be labelled as pseudo-Foster Kennedy Syndrome.

## Case presentation

**Figure 1:**
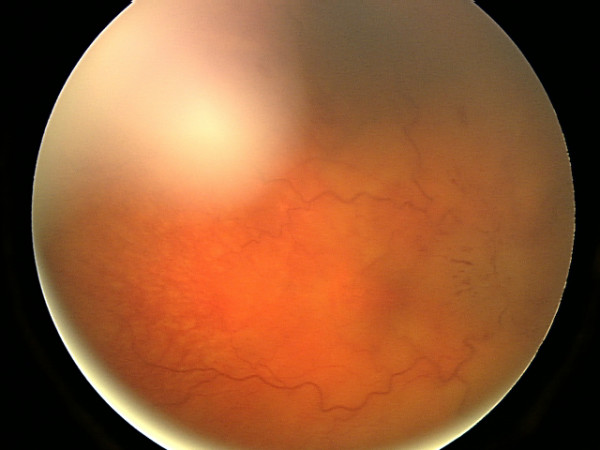
Fundal photograph showing severe papilloedema in the right eye.

**Figure 2:**
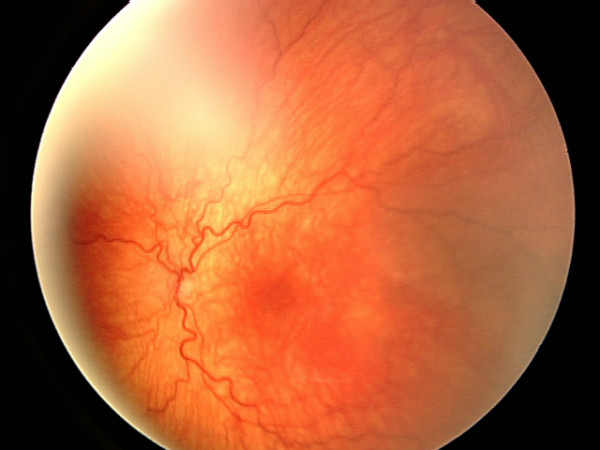
Fundal photograph showing a hypoplastic optic disc in the left eye.

A two year old boy with panhypopituitarism, hydrocephalus, developmental delay and obesity was referred for an ophthalmic opinion regarding concerns of recent severe visual deterioration. There were no other symptoms elicited of possible raised intra-cranial pressure. Magnetic resonance imaging showed Chiari malformation, ventricular dilatation and a small pituitary gland. Bedside fundoscopy was very difficult as the child kept moving. Prior to this presentation there was no documentation of baseline visual function. An examination under anaesthetic was performed and right-sided severe papilloedema and a hypoplastic left optic disc were found (Figures 1 and 2). The findings were indicative of raised intracranial pressure and the patient was urgently managed by the neurosurgeons with a ventriculoperitoneal shunt operation.

## Discussion

Hypoplastic optic disc is a congenital abnormality which may be unilateral or bilateral and is a characterised by a reduced diameter of the optic nerve head. Although clinically distinct from optic atrophy, it has been suggested that it is merely a type of non progressive optic atrophy acquired before the full development of the eye [1].

The appearance of unilateral optic disc swelling with contralateral optic disc atrophy has been described as the Foster Kennedy Syndrome. In "true" Foster-Kennedy Syndrome unilateral disc swelling is caused by a tumour on the inferior surface of the frontal lobe, compressing the optic nerve on one side with papilloedema contralaterally [2]. In the absence of an intracranial mass these findings may be labelled as pseudo-Foster Kennedy Syndrome, typically due to bilateral sequential optic neuritis or ischaemic optic neuropathy [3,4].

Explanations for the unilateral disc swelling in Foster Kennedy syndrome include failure of transmission of the intracranial pressure to the optic disc secondary to pressure on the vaginal sheath; or closure of the vascular bed of the optic disc [5]. Our case demonstrates that this finding may be observed in patients with unilateral optic disc hypoplasia and is thus another differential cause of pseudo-Foster Kennedy Syndrome.

## Conclusion

In this case the finding of unilateral papilloedema was due to a congenital abnormality of the left optic disc, preventing transmission of the raised intracranial pressure to the optic nerve head. This is important to bear in mind when examining children with optic nerve hypoplasia.

## Competing interests

The author(s) declare that they have no competing interests.

## Authors' contributions

SB was the lead author involved in carrying out the literature search, study design and writing the case report. TD assisted with writing the paper, supervising and managing the case. VL supervised the management of the case and participated in its design and approval. All authors have been involved in approving the final manuscript.

## Consent

The authors obtained written informed consent from the parents of this patient for the publication of this case report along with images. A copy of the written consent is available for review by the Editor-in-Chief of this journal.
